# Experimental performance study on alkali-activated coal gangue-slag gel stabilized spoil for road base preparation

**DOI:** 10.1371/journal.pone.0343272

**Published:** 2026-03-31

**Authors:** Xinyu Li, Jianan Sun, Yongjie Ding, Haoming Wang, Wei Wei

**Affiliations:** 1 Heilongjiang Communications Investment Highway Construction & Investment Co., Ltd., Harbin, China; 2 Chongqing Jiaotong University, Chongqing, China; 3 Faculty of architecture, civil and transportation engineering, Beijing University of Technology, Beijing, China; Ziane Achour University of Djelfa: Universite Ziane Achour de Djelfa, ALGERIA

## Abstract

This study developed a fully waste-based stabilized aggregate for road bases. A high-performance alkali-activated binder was synthesized from coal gangue and slag, then blended with tunnel-excavated spoil. Performance was compared to cement-stabilized spoil. The optimal mix had a slag-to-gangue ratio of 1:1, a sodium-silicate modulus of 0.8, a liquid-to-solid ratio of 0.38, and 14% alkali-activator content. Under this design, the binder reached 28-day compressive and flexural strengths of 46.2 MPa and 6.9 MPa, respectively. When used for spoil stabilization, the AA-GS system showed early-age strength benefits. UCS values reached 8–9 MPa across subbase dosages. Compared to cement-stabilized spoil, the AA-GS material reduced 90-day drying-shrinkage strain by 57.6%, water-loss rate by 23.4%, and shrinkage coefficient by 27.7%. The 28-day water-stability coefficient was no less than 0.876, and the freeze-thaw durability index remained at or above 0.80. These results demonstrate the AA-GS system offers strong mechanical performance and durability while enabling full reuse of tunnel spoil, coal gangue, and slag. The system was also designed with field applicability in mind, ensuring scalability and enabling real-world reuse of waste materials with tangible carbon reduction benefits.

## 1 Introduction

Direct landfilling or indiscriminate dumping of untreated tunnel-excavated spoil depletes land resources and triggers ecological risks and related environmental hazards [1–3]. Reported impacts include landslides and groundwater contamination [4,5], air pollution from dust during storage and transportation [6], and ecosystem disruption with habitat degradation around stockpiles [7]. At the same time, shortages of natural aggregates have been increasingly documented. Against this backdrop, mechanically robust tunnel-excavated rock fragments have been verified as suitable materials for base layer applications [8,9]. Riviera etal. characterized seven Alpine tunnel spoil types and demonstrated exceptional infrastructure performance [10]. Deng etal. optimized cement-stabilized mixtures containing recycled tunnel aggregates, achieving verified maximum dry density (2.25 g/cm^3^) and 6.8% optimum moisture content, supporting their road-construction potential [11]. Nevertheless, pre-processed tunnel spoil often requires cement stabilization for structural adequacy even when size specifications are met through screening and crushing [12,13].

Cement production has been recognized as a highly energy-intensive process with substantial carbon emissions. Approximately 0.44 tons of CO_2_ are released per ton of cement produced, contributing to 7% of global emissions [14,15]. These figures align with global carbon-neutrality goals [16] and motivate alkali-activated materials (AAMs) as low-carbon binders: by avoiding high-temperature calcination, AAMs reduce energy demand and CO_2_ emissions while offering competitive mechanical performance and chemical resistance [17–19].This rationale is supported by prior studies under ambient curing: alkali-activated slag-brick powder binders achieved a 7-day compressive strength of 67.29 MPa [20]; sodium-hydroxide/sodium-silicate-activated fly-ash-slag composites reached 40.68 MPa at 7 days [21]; MgO-NaOH activation of fly ash attained 34 MPa at 28 days [22]; and ground rice-husk-ash systems produced 40 MPa within 1 day of curing [23]. Microstructural evidence further shows increased hydration products in sodium-silicate-activated slag-copper-slag matrices [24].

The incorporation of AAMs into road base structures, combined with tunnel-excavated spoil as aggregates, establishes a dual solid-waste route that replaces conventional cement and natural aggregates while enabling high-value reuse of both streams [9,25]. For tunnel spoil stabilization, a 3:1 lime-phosphogypsum blend achieved optimal mechanical performance and water stability in subgrade applications [26]. Mixtures with 4% carbide slag, 4% phosphogypsum, and 4% red mud showed maximal reinforcement efficiency [27]. Geosynthetic-stabilized tunnel sludge, used for base/subbase layers, improved performance by reducing permanent deformation and by extending service life [28]. A 6:2 carbide slag-soda residue blend with lime met medium-traffic subgrade requirements [29]. Clinker-fly ash-gypsum gel-reinforced road bases delivered higher strength and reduced shrinkage cracking versus cement-stabilized bases [30]. Collectively, these outcomes highlight synergy between AAM binders (cohesion) and tunnel-spoil aggregates (skeletal reinforcement) that densifies the matrix and controls shrinkage, yielding dense, mechanically robust, eco-friendly, and cost-effective road-base materials for modern practice [31,32].

Beyond AAM-poil systems, complementary studies on sustainable binders and aggregates provide convergent evidence and practical design cues. Plastic (10%) or rubber (5%) with 3% cement raised CBR (~50% and ~28%), enabling ~435 mm thinner pavements [33]. In pervious concrete, 0–50% lightweight pumice aggregate (LWA) or waste glass granular (WGG) improved strength and permeability; LWA showed lower abrasion resistance than WGG [34]. Foamed concrete: fine fly ash lowered modulus (~20%) but cut shrinkage (~35% at 30%); small polypropylene fiber raised modulus (14–66%); typical E ≈ 1–8 GPa [35]. In heritage-building envelopes, silica-aerogel composites improved insulation, moisture control, and fire performance in pilot projects, though practical barriers persist [36]. Together, these findings frame the broader sustainability landscape and inform the present work’s material selection and performance targets.

In this study, we addressed the lack of standardized head-to-head benchmarks for alkali-activated binders by comparing an AA-GS-stabilized tunnel spoil with a cement-stabilized control under identical protocols. We evaluated unconfined compressive strength, drying shrinkage, water stability, and freeze-thaw resistance to quantify strength development, moisture resilience, and crack-resistance. We then positioned the outcomes against other binder systems reported in prior studies to clarify AA-GS’s (Abbreviations have been provided in Supplementary Information S1 File) relative performance. Finally, we outlined a dual-waste utilization pathway that coupled tunnel spoil with industrial by-products to deliver a practical, low-carbon, high-performance road-base solution.

## 2 Materials

### 2.1 Alkali-activated gangue-slag

**Fig 1:**
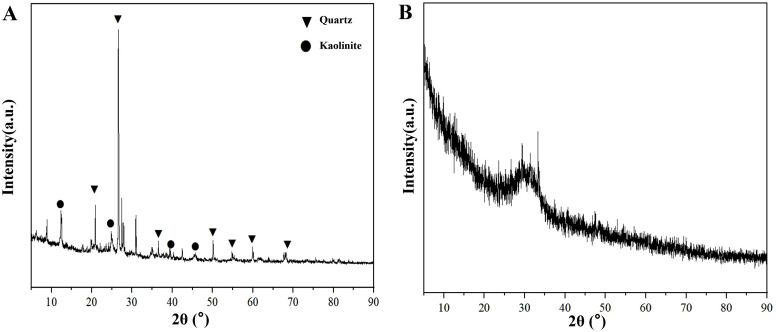
The results of laser particle size testing.

**Fig 2:**
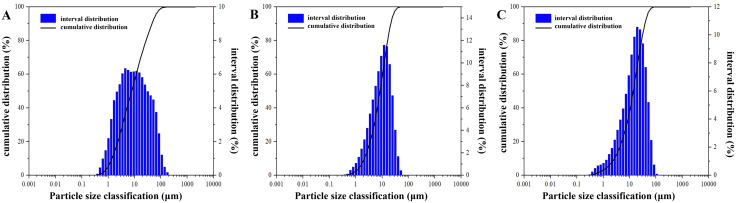
XRD Analysis Results.

The alkali-activated gangue-slag (AA-GS) gel utilized coal gangue sourced from Anada Mineral Powder Co., Ltd. (Shijiazhuang, Hebei Province), with X-ray fluorescence (XRF) analysis confirming SiO₂, Al₂O₃, and Fe₂O₃ as primary oxides alongside trace organic compounds and sulfides (Table 1). Laser granulometry measured a particle size range of 0.38–174.40 μm (mean: 21.31 μm; Fig 1). X-ray diffraction (XRD) patterns confirmed quartz and kaolinite as dominant crystalline phases (Fig 2). Blast furnace slag, supplied by Hebei Yousheng Mineral Co., Ltd., exhibited CaO and SiO_2_ predominance via XRF (Table 1), with particle sizes spanning 0.40–65.59 μm (mean: 13.25 μm; Fig 1). XRD analysis revealed a broad amorphous-phase hump near 30° (Fig 2), signifying high hydraulic reactivity in the glassy matrix.

**Table 1:** XRF Analysis Results.

components	SiO_2_	Al_2_O_3_	Fe_2_O_3_	CaO	MgO	Na_2_O	K_2_O	SO_3_	TiO_2_	others
gangue	64.17	26.89	2.12	0.89	0.79	1.13	1.72	0.43	0.83	1.03
slag	32.43	15.10	0.28	39.84	6.70	0.42	0.47	2.74	1.09	0.21

The alkali activator was composed of sodium silicate solution and sodium hydroxide pellets. The sodium silicate solution, supplied by Tianjin Zhonglian Chemical Reagent Co., Ltd., comprised 8.5% Na₂O, 27.0% SiO₂, and 64.5% H₂O by mass. Sodium hydroxide pellets (≥98% purity) were procured from Shandong Keyuan Biochemical Co., Ltd. Chemical compositions of both activator precursors are detailed in Table 2.

**Table 2:** Precursors of alkali activators.

Raw material	NaOH	Na₂O	SiO₂	H₂O	Form
Sodium hydroxide	≥98%	0	0	0	White solid pellets
Sodium silicate solution	0	8.5%	27.0%	64.5%	Translucent liquid

In practice, required sodium-silicate moduli vary across applications, and the water already contained in the sodium-silicate solution must be counted as mixing water. As a result, the modulus design and the total mixing water must be determined separately, which complicates dosage calculations. To streamline this process, we use Equations (1) and (2). Plain-language guide to Equations (1) and (2). Equation (1) computes the required NaOH mass once the target activator dosage, the as-supplied sodium-silicate composition, and the target modulus are set; it ensures the designed alkali content/modulus without trial-and-error. Equation (2) computes the extra mixing water after subtracting the water already carried by the sodium-silicate solution, so that the mixture meets the intended liquid-to-solid ratio (L/S). How to use (recipe). Step 1 Fix inputs (binder proportions, activator dosage, target modulus, L/S, and sodium-silicate composition). Step 2 Use Eq. (1) to obtain the NaOH mass and prepare the activator together with the measured sodium-silicate solution. Step 3 Use Eq. (2) to obtain the added water, ensuring the final mixture satisfies the target L/S.

Read-along symbols. ${\text{m}}_{\text{NaOH}}$ (g, Eq. 1 output); ${\text{m}}_{\text{CG}}$, ${\text{m}}_{\text{GGBS}}$ (g, binder inputs); ${\text{M}}_{{\text{N}\text{a}}_{\text{2}}\text{O}}=\text{61}.\text{99}$ (g mol ⁻ ¹, Eq. 1); ${\text{m}}_{{\text{H}}_{\text{2}}\text{O}}$ (g, Eq. 2 output); $\omega_{\text{alkali activators}}$ and $\omega_{{\text{H}}_{\text{2}}\text{O}}$ (mass %, sodium-silicate Na₂O and H₂O, Eqs. 1–2); k (target modulus, Eq. 1); L/S (design input, Eq. 2).


$${\text{m}}_{NaOH}\text{\textasciitilde{}=\textasciitilde{}}\frac{(m_{\text{CG}}+m_{GGBS})\times w_{alkali\cdot activators}}{\frac{M_{modulus}\times M_{Na2O}}{80W_{Na2O}\left(M_{modulus}-M{\text{'}}_{modulus}\right)}+1}$$ (1)

$$m_{H2O}=\left(m_{CG}+m_{GGBS}\right)\times \left(wH2O-\frac{M{\text{'}}_{modulus}\times M_{Na2O}\times w_{alkali\cdot activators}}{M{\text{'}}_{modulus}\times M_{Na2O}+80W_{Na2O}\left(M_{modulus}-M{\text{'}}_{modulus}\right)}\right)$$ (2)

The slag:coal gangue ratio of 1:1 was selected, with a sodium silicate modulus of 0.8, liquid-to-solid ratio of 0.38, and 14% alkali activator dosage, to formulate the alkali-activated coal gangue-slag cementitious material. Before testing, these parameters were fixed a priori to satisfy field-oriented constraints. We targeted a flow of approximately 170 ± 10 mm under ambient curing. We aimed for an initial setting time between 90 and 150 min. We set a 28-day compressive-strength threshold of at least 45 MPa. To balance workability and reactivity under these constraints, we adopted a 1:1 slag–gangue ratio. A high slag fraction increased viscosity, alkali demand, and shrinkage risk. A high gangue fraction reduced early reaction. We chose a 14% activator as the minimum that met the strength and durability targets. Higher dosages provided only small strength gains but increased efflorescence and shrinkage, whereas lower dosages under-activated the gangue. We set the silicate modulus to 0.8 and the liquid-to-solid ratio to 0.38 to control viscosity and compaction at ambient temperature. These inputs aligned with the mass-balance calculations in Equations (1) ad (2). Under this mix design, the AA-GS gel showed excellent fluidity, appropriate setting time, and high compressive and flexural strengths (Table 3).

**Table 3:** Performance evaluation of AA-GS gel under optimized mix proportion.

Property	3d	7d	28d
Compressive strength (MPa)	35.4	39.9	46.2
Flexural strength (MPa)	3.6	5.3	6.9
Initial setting time (min)	96		
Final setting time (min)	141		
Fluidity (mm)	174		

### 2.2 P·O 42.5—Conventional binder and control group

The cement used in this study was Portland cement Grade 42.5 (P·O 42.5). Particle size distribution analysis (Fig 2) revealed a mean particle diameter of 22.52 μm and a specific surface area of 0.305 m^2^/g. As summarized in Table 4, all technical parameters of the cement were confirmed to comply with the Chinese National Standard for Common Portland Cement (GB 175–2007).

**Table 4:** Cement performance parameters.

Property		Measured value	Specification requirement
Fineness (%)		3.1	≤10
Soundness		qualified	qualified
Initial setting time (min)		157	≥45
Final setting time (min)		264	≤600
Compressive strength (MPa)	3d	23.6	≥17.0
	28d	45.1	≥42.5
Flexural strength (MPa)	3d	4.7	≥3.5
	28d	7.2	≥6.5

### 2.3 Aggregate from tunnel muck

The tunnel muck aggregates employed in the research were obtained from excavation materials of Taiqing Tunnel along the Hei-Yi Expressway in Heilongjiang Province. Based on particle size ranges, the aggregates were categorized into four size fractions: 0–4.75 mm, 4.75–9.5 mm, 9.5–19.0 mm, and 19.0–26.5 mm. Performance testing was conducted in accordance with the Test Methods for Highway Engineering Aggregates (JTG 3432–2024), with results presented in Table 5. The tunnel muck was confirmed to fulfill the utilization criteria for base course aggregates in expressways and Class-I highways specified in the Technical Specifications for Construction of Highway Pavement Base Layers (JTG/T F20—2015).

**Table 5:** Test results of tunnel muck aggregate properties.

Property	Particle size (mm)				
	19-26.5	9.5-19	4.75-9.5	0-4.75	Technical requirement
Apparent relative density (g/cm^3^)	2.734	2.739	2.741	2.787	≥2.65
Crushing value (%)	22			–	≤22
Flakiness content (%)	8.12	7.23	11.12	–	≤18
Water absorption (%)	0.43	0.71	2.31	2.78	Tested value
Weak particle content (%)	2.4		–	–	≤3
Dust content (<0.075 mm, %)	0.8	0.9	1.1	–	≤1.2
Plasticity index	–	–	–	12	≤17

To facilitate replication beyond laboratory batches, we implemented a minimal quality-control routine that stayed within the validated mix envelope (silicate modulus 0.8; activator 14% at the paste scale; L/S = 0.38; subbase binder 4–8% or activator 0.6–1.5%). Each incoming lot of coal gangue and slag was screened by XRF/XRD for CaO, SiO₂, Al₂O₃, Fe₂O₃ and SO₃; adjacent deliveries were pre-blended when oxide trends departed from the recent average. Powder fineness was kept within ±10% of the laboratory baseline. During batching, tunnel-spoil moisture was controlled around the Proctor OMC (±1–2% absolute) and field compaction targeted ≥98% of MDD. Activator solution followed the low-modulus route (0.8); solids content was checked by refractometry and the effective L/S was held within ±0.02 by metered water. All operations were conducted at ambient temperature; no heat curing or autoclaving was introduced.

## 3 Experiment

### 3.1 Flow performance experiment

#### 3.1.1 Mixing method of samples.

The AA-GS gel-stabilized tunnel muck material was mixed and shaped based on the static compaction method (T 0843–2009) specified in the *Test Specifications for Inorganic Binder-Stabilized Materials in Highway Engineering* (JTG/T F20–2015). The procedure was executed as follows. First, sodium hydroxide, sodium silicate, and 20% of the total mixing water were precisely weighed according to the designed mix ratio to prepare an alkali activator with the required modulus. Second, solid waste powders were weighed based on the optimal coal gangue-slag-geopolymer ratio, followed by dry mixing in a planetary mixer for 3 minutes. Third, aggregates and 80% of the total water were weighed according to gradation design and compaction test results, homogenized in a metal tray, and sealed in a plastic bag for curing (≤24 hours). Fourth, the pre-mixed solid waste powders and cured aggregates were combined in the mixer for 3 minutes, during which the alkali activator was incrementally added. Fifth, molds pre-greased with butter were filled with the mixture in 2–3 layers, each compacted uniformly with a tamping rod. Sixth, the molds were compressed in a press at 1 mm/min until the top and bottom plates fully engaged, with pressure maintained for 2 minutes. Seventh, after depressurization, the molds were removed, demolded after 2–4 hours, and the specimens were wrapped in film, cured in a chamber, and soaked for 24 hours before testing at designated ages (Table 6).

**Table 6:** Specimen dimensions.

Test category	Dimensions (mm)
Unconfined compressive strength	Φ150 × 150
Drying shrinkage	100 × 100 × 400
Water stability	Φ150 × 150
Freeze-thaw resistance	Φ150 × 150

### 3.2 Mix proportion design

#### 3.2.1 Gradation design.

**Fig 3:**
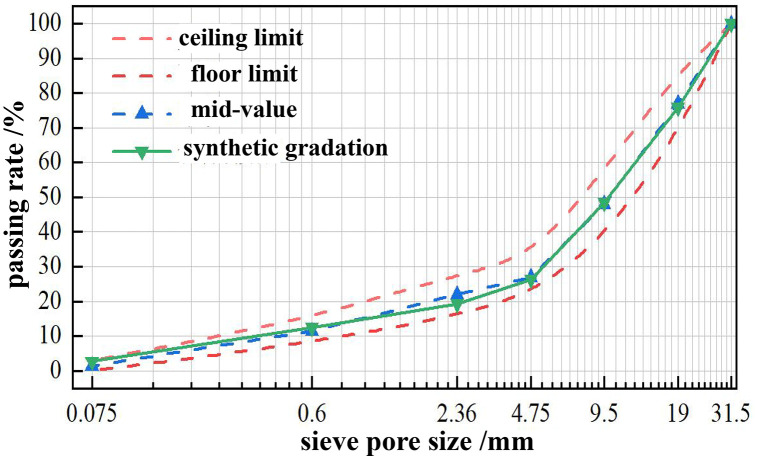
Synthetic Gradation Curve of Tunnel Muck Aggregate.

To ensure excellent strength, load-bearing capacity, and road performance of the stabilized base course, aggregate gradation in the mixed materials must be optimized. Gradation design for aggregates in the stabilized base was conducted in accordance with the C-B-3 gradation standard (Table 7) recommended by the *Technical Specifications for Construction of Highway Pavement Bases* (JTG/T F20—2015). The sieve analysis results of the target synthesized gradation were documented in Table 8, with corresponding gradation curves illustrated in Fig 3.

**Table 7:** C-B-3 Gradation.

Gradation	Passing rate (%) by mass for sieve sizes (mm)						
	31.5	19	9.5	4.75	2.36	0.6	0.075
Range	100	68-86	38-58	22-32	16-28	8-15	0-3

**Table 8:** Synthesized Gradation of Tunnel Muck.

Gradation Range (mm)	Proportion (%)	Passing rate (%) for sieve sizes (mm)						
		31.5	19	9.5	4.75	2.36	0.6	0.075
0-5	26	100.0	100.0	100.0	94.2	72.4	46.7	10.5
5-10	28	100.0	100.0	78.7	5.7	0.9	0.6	0.0
10-20	24	100.0	86.3	1.7	0.7	0.5	0.5	0.0
20-30	22	100.0	5.4	0.2	0.1	0.1	0.1	0.0
Upper limit		100.0	86.0	58.0	32.0	28.0	15.0	3.0
Lower limit		100.0	68.0	38.0	22.0	16.0	8.0	0.0
Median gradation		100.0	77.0	48.0	27.0	22.0	11.5	1.5
Synthesized gradation		100.0	75.9	48.5	26.3	19.2	12.4	2.7

#### 3.2.2 Mix proportion design of stable base course.

The stabilizer dosage range was controlled between 3% and 10% to meet subgrade bearing capacity specifications. The experimental design was applied in tunnel construction (Table 9), so five different dosages were selected for the mixture design. Using the mix proportions determined in Section 2.2, the dosages of each component in the experimental group were calculated using Equations 1 and 2, and the results are shown in Tables 10 and 11.

**Table 9:** Dosage Range of AA – GS gel.

Dosage of Solid Waste Powder (%)	Dosage Range of AA-GS gel (%)		
	Lower Limit	Upper Limit	Value
4	0.6	0.9	0.6, 0.7, 0.8
5	0.7	1.1	0.7, 0.8, 0.9
6	0.8	1.2	0.8, 0.9, 1.0
7	1.0	1.4	1.0, 1.1, 1.2
8	1.1	1.5	1.1, 1.2, 1.3

**Table 10:** Mix Proportions of AA – GS gel Solidified Base Course.

Number	Dosage of Solid Waste Powder (%)	Moisture Content (%)	Dosage of Alkali Activator (%)	Modulus	Solid Waste Powder (g)	Water Glass (g)	NaOH (g)	H_2_O (g)
FT-4–0.6	4%	8.9	0.6%	0.8	218.4	24.6	8.1	489.5
FT-4–0.7			0.7%			28.8	9.5	486.8
FT-4–0.8			0.8%			32.9	10.8	484.2
FT-5–0.7	5%	9.1	0.7%		271.4	28.6	9.4	500.2
FT-5–0.8			0.8%			32.7	10.8	497.6
FT-5–0.9			0.9%			36.8	12.1	494.9
FT-6–0.8	6%	9.5	0.8%		325.0	32.6	10.7	524.4
FT-6–0.9			0.9%			36.7	12.1	521.8
FT-6–1.0			1.0%			40.8	13.4	519.2
FT-7–1.0	7%	9.8	1.0%		376.8	40.5	13.3	538.4
FT-7–1.1			1.1%			44.6	14.7	535.8
FT-7–1.2			1.2%			48.6	16.0	533.2
FT-8–1.1	8%	10.1	1.1%		431.0	44.6	14.7	558.9
FT-8–1.2			1.2%			48.6	16.0	556.3
FT-8–1.3			1.3%			52.7	17.3	553.7

**Table 11:** Mix Proportions of the Cement-Solidified Base Course.

Serial Number	Cement Dosage (%)	Moisture Content (%)	Cement (g)	H_2_O (g)
CT-4	4	8.2	219.3	467.6
CT-5	5	8.9	271.8	508.0
CT-6	6	9.1	325.2	522.7
CT-7	7	9.6	376.5	552.5
CT-8	8	9.9	432.0	577.4

### 3.3 Unconfined compressive strength

The unconfined compressive strength of inorganic binder-stabilized materials was tested in accordance with Method T 0805−2024 of *Specifications for Test Methods of Inorganic Binder-Stabilized Materials in Highway Engineering* (JTG 3341−2024). Specimens cured underwater for 24 hours were removed from the tank, and surface moisture was eliminated by gentle wiping with a clean towel or absorbent cloth. Dried specimens were positioned at the center of the compression machine’s loading plate. Unconfined compressive strength testing was conducted at a constant displacement rate of 1 mm/min until failure occurred, with the maximum load at failure recorded. Calculations were performed using Equations (3) and (4). In Equation (3), (4), the unconfined compressive strength $R_{c}(\text{MPa})$ was calculated as $R_{c}=P/A$, where P(N) was the peak load at failure and $A({mm}^{\text{2}})$ was the specimen’s cross-sectional area given by $A=\pi (D/\text{2}{)}^{\text{2}}$; D(mm) denoted the specimen diameter.


$$R_{c}=\frac{P}{A}$$ (3)

$$A=\frac{\text{1}}{\text{4}}\pi D^{\text{2}}$$ (4)

### 3.4 Dry shrinkage performance test

The dry shrinkage of inorganic binder-stabilized materials was tested according to Method T 0854–2024 in *Specifications for Test Methods of Inorganic Binder-Stabilized Materials in Highway Engineering* (JTG 3341–2024). Beam specimens were prepared and cured under standard conditions for 7 days, with immersion in water during the final 24 hours of curing. After saturation, surface moisture was removed, and initial specimen length was measured at three distinct locations using a vernier caliper, with the average value calculated. Initial mass m_0_ was recorded once no visible moisture remained on the surface. A shrinkage device was prepared by grinding the specimen ends, bonding polymethyl methacrylate plates to the polished surfaces with cyanoacrylate adhesive, and securing a sensor clamp after adhesive solidification. Lubricant-coated glass rods were installed on the shrinkage device to minimize friction during contraction. The shrinkage device and specimens were placed in a curing chamber maintained at 20 ± 0.5°C and 60 ± 5% relative humidity. Specimen mass m_i_ and shrinkage displacement δ_i_ were measured and recorded at intervals from the time of chamber placement. Water loss rate, drying shrinkage strain, drying shrinkage coefficient, and total drying shrinkage coefficient were calculated using Equations (5)–(8). The time-indexed variables were defined as follows: the water-loss rate $w_{i}(\%)$ at the *i*-th reading; the dry-shrinkage displacement $\delta_{i}(\text{mm})$ at the *i*-th reading; the dry-shrinkage strain $\varepsilon_{i}(\mu \epsilon )$ at the *i*-th reading; the dry-shrinkage coefficient $\alpha_{\text{d}i}(\mu \epsilon /\%)$ at the *i*-th reading; and the total dry-shrinkage coefficient $\alpha_{\text{d}}(\mu \epsilon /\%)$. Mass- and geometry-related terms included the weighed mass of the standard specimen $m_{i}(\text{g})$ at the *i*-th reading, the specimen length l(mm), and the constant (oven-dry) mass $m_{\text{p}}(\text{g})$.


$$w_{i}=\frac{m_{i}-m_{i}+1}{m_{p}}$$ (5)

$$\varepsilon_{i}=\frac{\delta_{i}}{l}$$ (6)

$$\alpha_{di}=\frac{\varepsilon_{i}}{w_{i}}$$ (7)

$$\alpha_{d}=\frac{\sum \varepsilon_{i}}{\sum_{wi}}$$ (8)

### 3.5 Water stability test

The compressive strength of specimens cured under prolonged water immersion was tested and compared with the unconfined compressive strength of specimens under standard curing conditions. The water stability coefficient and strength loss rate were calculated using Equations (9) and (10), respectively. The water-stability coefficient $K_{R}(-)$ was calculated as $K_{R}=R_{\text{wc}}/R_{\text{sc}}$, where $R_{\text{wc}}(\text{MPa})$ denoted the unconfined compressive strength after immersion curing and $R_{\text{sc}}(\text{MPa})$ denoted the unconfined compressive strength after standard curing. The strength-loss rate ΔR(%) was computed as $\Delta R=(R_{\text{sc}}-R_{\text{wc}})/R_{\text{sc}}\times \text{1}\text{0}\text{0}\%$.


$$K_{R}=\frac{R_{wc}}{R_{sc}}$$ (9)

$$\Delta R=\frac{R_{sc}-R_{wc}}{R_{sc}}\times 100\%$$ (10)

### 3.6 Freeze-thaw cycle resistance test

The freeze-thaw resistance of alkali-activated cement-stabilized tunnel slag base mixtures was evaluated according to Test Method T 0858–2009 for inorganic binder-stabilized materials in the *Test Specifications for Inorganic Binder-Stabilized Materials in Highway Engineering* (JTG 3341–2024). Specimens were saturated for 24 h on the final day of curing, with a water level maintained approximately 25 mm above the specimen surface. Specimen masses were measured before saturation. Following saturation, specimens were removed, surface moisture was wiped, and they were placed in a low-temperature chamber set to −18 °C for 16 h of freezing. After freezing, specimens were extracted, reweighed, and immediately submerged in a 20 °C water bath for 8 h of thawing. Specimens were dried, reweighed, and one freeze-thaw cycle was completed. Specimens cured for 28 days underwent five cycles, while those cured for 90 days underwent ten cycles. Detailed test parameters are provided in Table 12. The frost resistance index and mass loss rate of the specimen are calculated according to Formulas 11 and 12 respectively. The compressive-strength change ratio after *n*freeze–thaw cycles BDR(−, %) was calculated as $\text{BDR}=(R_{\text{dc}}-R_{c})/R_{c}\times \text{1}\text{0}\text{0}\%$, where $R_{\text{dc}}(\text{MPa})$ denoted the compressive strength of the specimen after *n*freeze–thaw cycles and $R_{c}(\text{MPa})$ denoted the compressive strength of the reference specimen. The mass-change rate after *n*freeze–thaw cycles $W_{n}(-,\;\%)$ was computed as $W_{n}=(m_{n}-m_{\text{0}})/m_{\text{0}}\times \text{1}\text{0}\text{0}\%$, where $m_{\text{0}}(\text{g})$ and $m_{n}(\text{g})$ were the specimen masses before the cycles and after the *n*-th cycle, respectively.

**Table 12:** Parameters of Frost Resistance Test.

Specimen Type	Specimen Size (mm)	Curing Age (d)	Number of Freeze-Thaw Cycles
Cylindrical	Φ150 × 150	28	5
		56	10


$$BDR=\frac{R_{dc}}{R_{c}}\times 100$$ (11)

$$Wn=\frac{m_{0}-m_{n}}{m_{0}}\times 100$$ (12)

### 3.7 XRF Experiment

The chemical compositions of three materials were determined using a PANALYTICAL AXIOS X-ray fluorescence spectrometer. The instruments were purchased from Guangzhou Jingu Scientific Instruments Co., Ltd. Ambient temperature was maintained at 10–35 °C with a stability of ±0.5 °C. Power supply voltage was set to 220 V ± 10%, and output fluctuations were restricted to within ±0.005%.

### 3.8 XRD experiment

Cut ceramsite specimens (10 × 20 mm) were analyzed for mineralogy and chemical reactions using a D8 Advance X-ray diffractometer. The instruments were purchased from Shanghai Aiyitong Network Technology Co., Ltd. Scans were performed at 40 mA and 40 kV, with a 2θ range of 5°–70° and a step size of 0.02° 2θ.

### 3.9 Laser particle size experiment

Particle size distribution of silt was measured via wet dispersion using a Mastersizer 3000 instrument. The instruments were purchased from Shenzhen Pugui Technology Co., Ltd. Ethanol was selected as the dispersion medium, covering a particle size range of 0.04–2000 μm.

### 3.10 SEM experiment

The study used a GeminiSEM 300 field emission scanning electron microscope to observe the microstructure. The instruments were purchased from Jiaxing Wanju International Supply Co., Ltd. The position parameters include an X/Y-axis travel of 130 mm, a Z-axis travel of 50 mm, and an inclination range of 20°.

## 4 Results

### 4.1 Analysis of unconfined compressive strength

**Fig 4:**
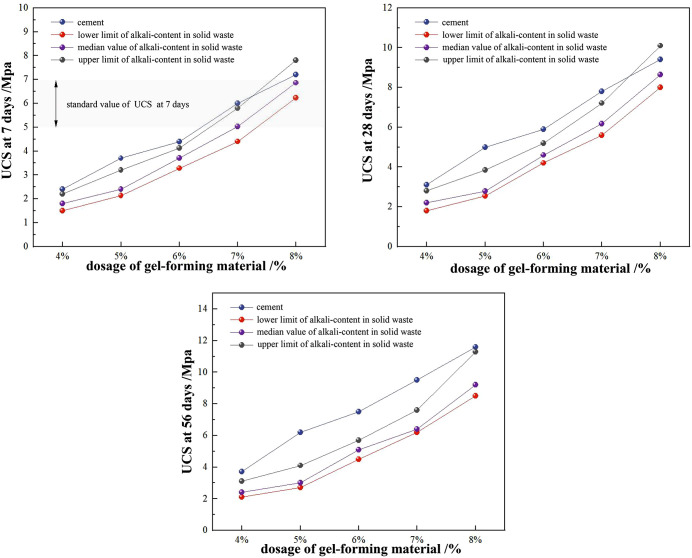
Influence of Changes in Cementitious Materials and Alkali Dosage on Unconfined Compressive Strength at Different Ages.

The unconfined compressive strength test results of alkali-activated cement-stabilized tunnel slag (FT) and cement-stabilized tunnel slag (CT) at 7-, 28-, and 56-day curing ages are presented in Table 13. To further analyze the influence of increasing binder and alkali activator contents on the unconfined compressive strength of FT, variations in strength under different binder–alkali ratios and curing periods were plotted (Fig 4). Both FT and CT exhibited progressive strength enhancements with higher binder content. This behavior was attributed to increased formation of hydration products following alkali activation or cement hydration reactions. These products were observed to coat tunnel slag surfaces, infiltrate aggregate pores, and enhance interparticle bonding, improving structural density and strength [37–40]. Consequently, the compressive strength of both materials demonstrated an increasing trend with extended curing durations, although the pace and timing of strength gain differed between FT and CT.

**Table 13:** Test Results of Unconfined Compressive Strength of Tunnel Muck Stabilized by Alkali-activated and Cementitious Binder.

Serial Number	Compressive Strength at 7 days(MPa)	Compressive Strength at 28 days(MPa)	Compressive Strength at 56 days (MPa)	Growth Rate from 7 days to 28 days (%)	Growth Rate from 28 days to 56 days (%)
CT-4	2.4	3.1	3.7	29.2	19.4
CT-5	3.7	5.0	6.2	35.1	24.0
CT-6	4.4	5.9	7.5	34.1	27.1
CT-7	6.0	7.8	9.5	30.0	21.8
CT-8	7.2	9.4	11.6	30.6	23.4
FT-4–0.6	1.5	1.8	2.1	20.0	16.7
FT-4–0.7	1.8	2.2	2.4	22.2	9.1
FT-4–0.8	2.2	2.8	3.1	27.3	10.7
FT-5–0.7	2.1	2.5	2.7	18.7	6.3
FT-5–0.8	2.4	2.8	3.0	15.8	7.9
FT-5–0.9	3.2	3.8	4.1	20.0	6.8
FT-6–0.8	3.3	4.2	4.5	27.9	7.1
FT-6–0.9	3.7	4.6	5.1	24.0	10.9
FT-6–1.0	4.1	5.2	5.7	26.0	9.6
FT-7–1.0	4.4	5.6	6.2	27.2	10.7
FT-7–1.1	5.0	6.2	6.4	22.6	3.7
FT-7–1.2	5.8	7.2	7.6	24.3	5.4
FT-8–1.1	6.2	8.0	8.5	28.4	6.3
FT-8–1.2	6.9	8.6	9.2	25.9	6.5
FT-8–1.3	7.2	9.2	10.2	27.6	10.9

**Fig 5:**
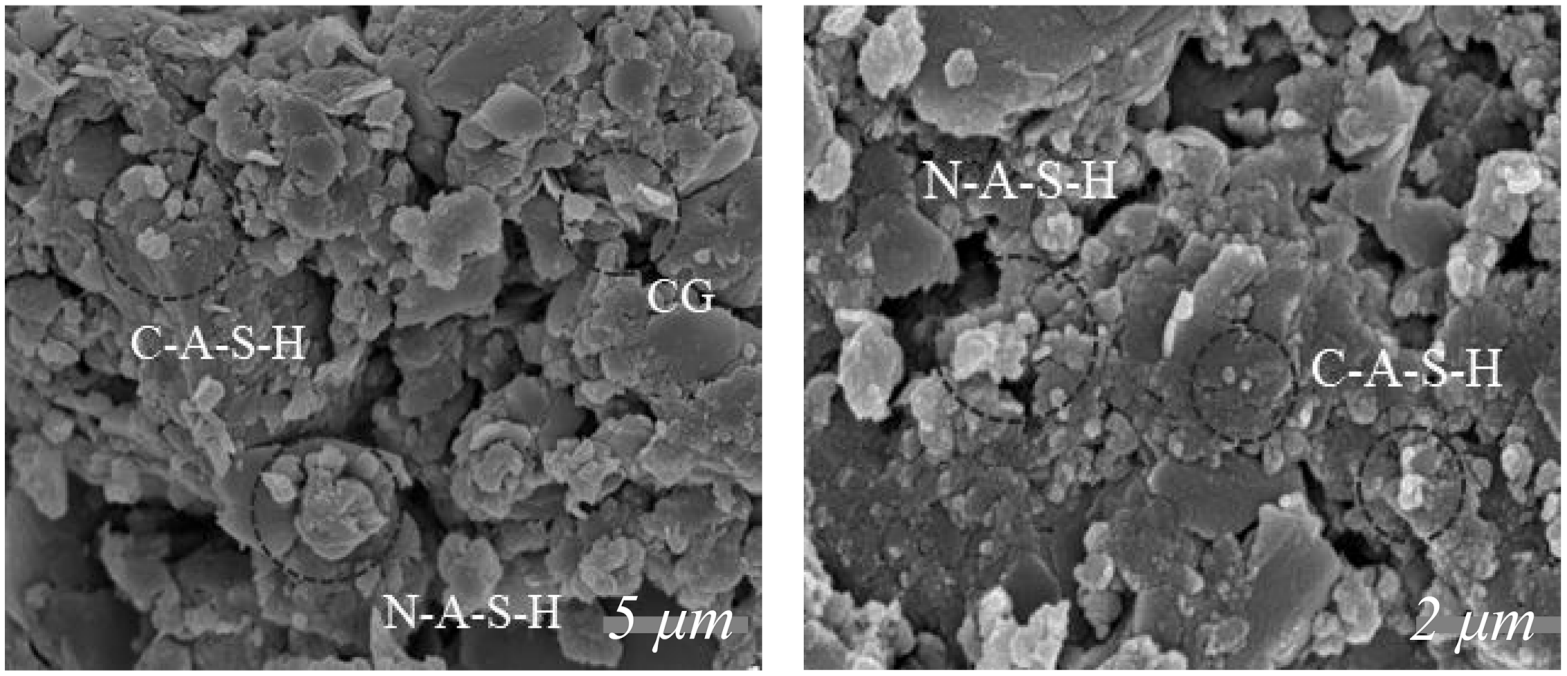
SEM test results.

FT displayed rapid strength development during the initial 7-day period, with most of the final capacity realized by 28 days and only incremental gains thereafter. In contrast, CT developed more gradually at early ages and showed a more pronounced increase by 28 days, with continued development afterward. The difference in strength growth between the two stabilizing materials mainly stemmed from their different reaction mechanisms (Fig 5). SEM observations showed that FT formed continuous C-(A)-S-H/N-A-S-H gel films that bridged slag and gangue particles, along with clear reaction rims around partially reacted grains and a compact interfacial transition zone (ITZ) containing fewer intergranular voids than CT. From 7 to 28 days, progressive gel infilling narrowed capillary necks and increased tortuosity, enabling more efficient load transfer at a modest overall degree of reaction and thereby explaining the accelerated early-age UCS development of FT. The same densification pathway reduced the number of percolating moisture pathways and strengthened particle–matrix bonding, which aligned with the higher water-stability coefficients and lower post-immersion strength loss observed for FT. By shifting the critical pore-size distribution toward smaller sizes and reinforcing the ITZ, FT also limited ice formation in vulnerable pores and lowered thaw-induced hydraulic pressures, consistent with the higher freeze–thaw durability coefficients and lower mass variation. Finally, the less-connected capillary network restrained moisture mobility, which cohered with the lower drying-shrinkage indices of FT compared with CT.

The FT material was governed by fast alkaline dissolution and early gel precipitation that bridged particles and tightened the interfacial transition zone (ITZ) [41–43]. Over 7–28 days, continuing gel growth in FT filled capillary pores and shifted the critical pore-size distribution toward smaller sizes, improving load transfer at a lower overall degree of hydration [44,45]. In comparison, the cement material primarily underwent further hydration of C3S, generating more C-S-H gels, which improved density and reduced porosity, resulting in a noticeable strength increase at 28 days [46,47]. In the later stage (28–56 days), the alkali-activated reactions gradually approached equilibrium, so strength increased only slightly, whereas cement hydration continued through delayed C3S hydration, enabling steady strength increases even after 56 days [47,48].

Elevated alkali activator dosages in FT consistently increased strength at all curing ages. This trend was associated with improved dissolution of reactive ions from solid-waste powders, enhanced gel formation, and increased microstructural density [49,50]. Higher alkali concentrations facilitated earlier gel generation and pore filling, improving mechanical performance [51,52]. As shown in Fig 4, several mixes met the 7-day design threshold for heavy-traffic bases. Considering cost, carbon, and shrinkage penalties at high alkali, five mixes (CT-7, FT-7–1.1, FT-7–1.2, FT-8–1.1, and FT-8–1.2) were selected for subsequent drying-shrinkage testing. Several FT mixes satisfied the 7-day UCS requirement for heavy-traffic base layers specified in JTG/T F20-2015 and were therefore advanced to shrinkage and durability evaluation.

### 4.2 Analysis of dry shrinkage performance

The data on dry shrinkage amount obtained from the dry shrinkage test were presented in S2 File, the data on dry shrinkage strain in S3 File, the data on water loss rate in S4 File, and the data on total dry shrinkage coefficient in S5 File.

**Fig 6:**
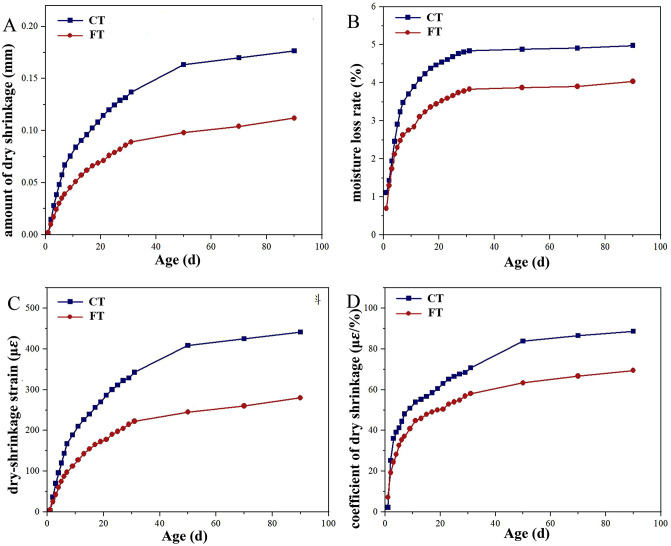
Analysis of the drying shrinkage results.

Fig 6 showed the development curves of drying shrinkage, water loss rate, shrinkage strain, and shrinkage coefficient for alkali-activated cement-stabilized tunnel slag (FT) and cement-stabilized tunnel slag (CT) over curing time. As shown in Fig 6, the water loss rate, shrinkage magnitude, strain, and coefficient for both materials each progressively increased with curing age, exhibiting rapid growth during the initial 10 days, gradual deceleration between 10 and 30 days, and stabilization beyond 30 days. This trend reflected elevated internal moisture content and porosity during the early curing phase (0–10 days), which facilitated water evaporation, together with limited gel formation and weak restraint on internal deformation, leading to accelerated shrinkage [53,54]. As hydration and alkali-activated reactions progressed, capillary pores became increasingly filled with gel, the pore structure became refined, and moisture migration pathways were further reduced. Additionally, declining internal relative humidity during hardening initially increased the rate of moisture loss, followed by slower water migration as humidity decreased, resulting in moderated increases in water loss and shrinkage coefficients between 10 and 30 days [55]. By 30 days, hydration and alkali activation were largely completed, internal humidity stabilized, and a dense microstructure had formed, contributing to stabilized shrinkage behavior.

FT showed significantly lower shrinkage strain, water loss rate, and shrinkage coefficient compared to CT at 90 days. This behavior reflected denser gel networks, finer pore sizes, and fewer continuous moisture pathways in the alkali-activated system, which together reduced capillary tension and volumetric contraction [56,57]. From a practical standpoint, FT therefore presented a lower cracking risk under dry and hot conditions.

**Fig 7:**
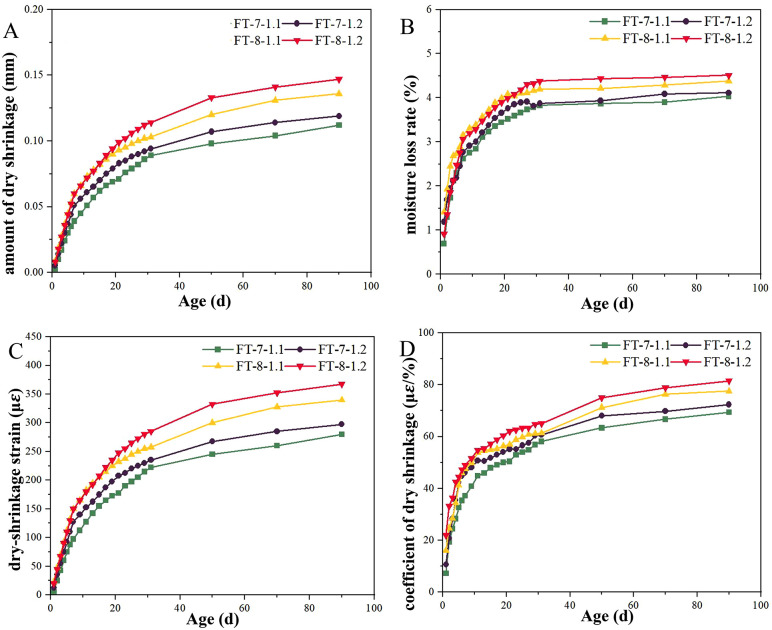
The changes of dry shrinkage under different dosages.

Fig 7 illustrated the development of drying shrinkage, water loss rate, shrinkage strain, and shrinkage coefficient for FT mixtures under varying dosages of alkali-activated binder and alkali activator across curing ages. As indicated in Fig 7, all four indices increased when either the binder or the activator content was raised. Enhanced binder and activator dosages tended to intensify hydration and alkali-activated reactions, increasing dehydration-induced contraction; at higher dosages, viscosity rose and residual air voids were more likely, enlarging the effective pore network [58–60]. Furthermore, higher binder dosages required elevated optimum moisture content, which accelerated water loss and pore formation, with capillary tension from fine-pore dehydration further amplifying shrinkage [61,62]. Design implication: to balance compaction, strength, and shrinkage, mixtures should minimize binder and activator while meeting structural requirements, with priority given to liquid-to-solid control and early pore refinement rather than simply increasing alkali. The FT mixtures kept the 90-day shrinkage indices within the allowable limits for semi-rigid bases under JTG/T F20-2015 and remained lower than the CT counterparts.

### 4.3 Analysis of water stability performance

**Fig 8:**
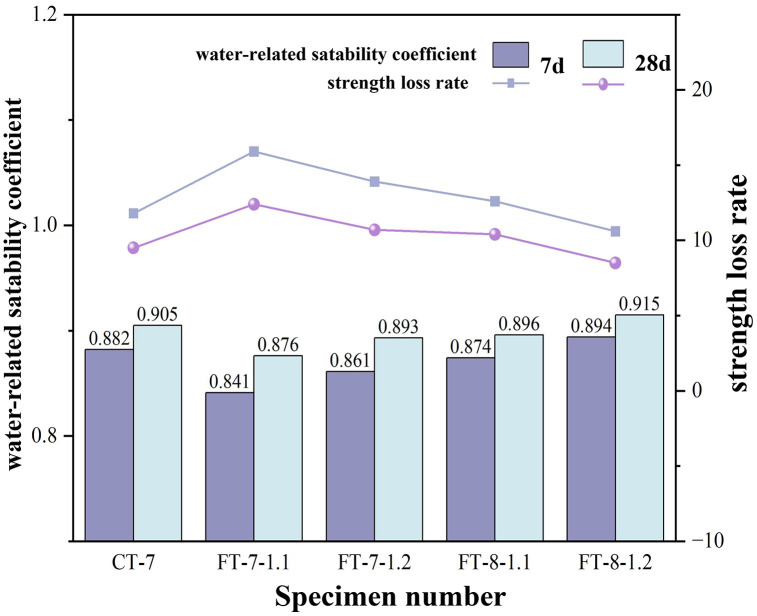
The water stability coefficient and strength loss rate of each specimen at different ages.

Materials with robust water stability effectively resist water erosion and hydrolysis, maintaining the structural integrity of base layers. The study evaluated water stability by monitoring strength variations in semi-rigid base materials after water immersion curing. Results from FT and CT water stability tests at different curing ages are summarized in Table 14. Increasing either the binder content or the activator dosage improved water-stability coefficients and reduced strength-loss rates at both 7 and 28 days (Fig 8; Table 14). The improvement was more pronounced at higher binder contents, indicating that additional reaction products enhanced pore blocking and barrier properties. Mechanistically, intensified alkali-activated reactions generated stable gels that filled pores, refined the pore-size distribution, and closed transport pathways, thereby lowering permeability and mitigating moisture-induced damage.

**Table 14:** Results of the 7-day Water Stability Test.

Serial Number	Strength after Immersion Curing (MPa)		Strength after Standard Curing (MPa)		Water Stability Coefficient		Strength Loss Rate (%)	
*-day	7-day	28-day	7-day	28-day	7-day	28-day	7-day	28-day
CT-7	5.3	7.1	6.0	7.8	0.882	0.905	11.8	9.5
FT-7–1.1	4.2	5.3	5.0	6.1	0.841	0.876	15.9	12.4
FT-7–1.2	5.0	6.4	5.8	7.2	0.861	0.893	13.9	10.7
FT-8–1.1	5.4	7.2	6.2	8.0	0.874	0.896	12.6	10.4
FT-8–1.2	6.2	7.9	6.9	8.6	0.894	0.915	10.6	8.5

Additionally, curing age played a positive role: by 28 days the mixtures generally exhibited higher water-stability coefficients and lower strength-loss rates than at 7 days, consistent with continued gel growth and capillary necking that further reduced permeability. Engineering implication: for moisture-sensitive subbases, gains in water stability were best achieved by modest increases in binder coupled with sufficient activator to close transport pathways; further increases in activator alone delivered diminishing returns and could heighten shrinkage risk. At 28 days, the water-stability coefficients were no less than the specification’s lower bound for base/subbase materials, demonstrating compliance with JTG/T F20-2015.

### 4.4 Analysis of freeze-thaw performance

Freeze–thaw strength deterioration in semi-rigid base materials primarily originated from ice-expansion–induced pore-water frost heave, generating internal stresses that compressed and damaged pore walls. Compressive strength was measured after five and ten freeze–thaw cycles on specimens cured for 28 and 56 days, and freeze–thaw coefficients were calculated to compare frost resistance between alkali-activated and cement-stabilized mixtures.

**Fig 9:**
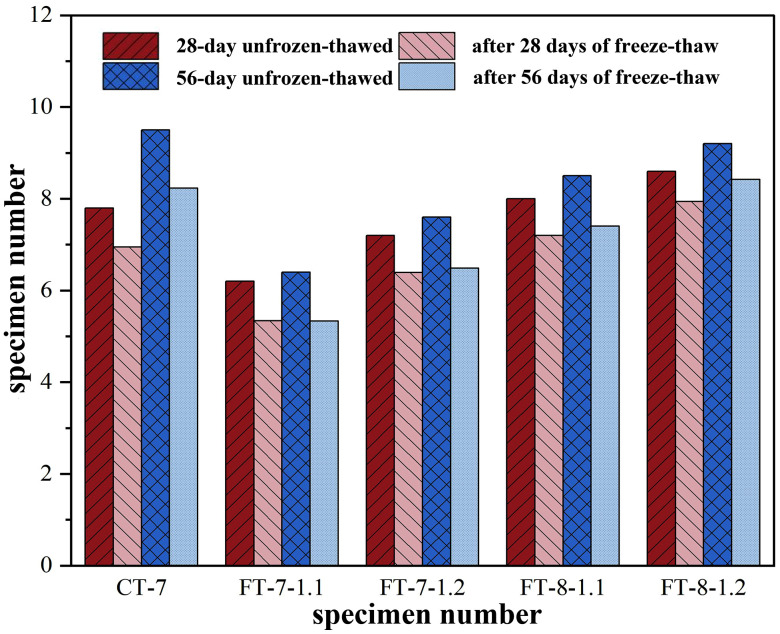
Changes in Compressive Strength under Freeze-Thaw Cycles.

**Fig 10:**
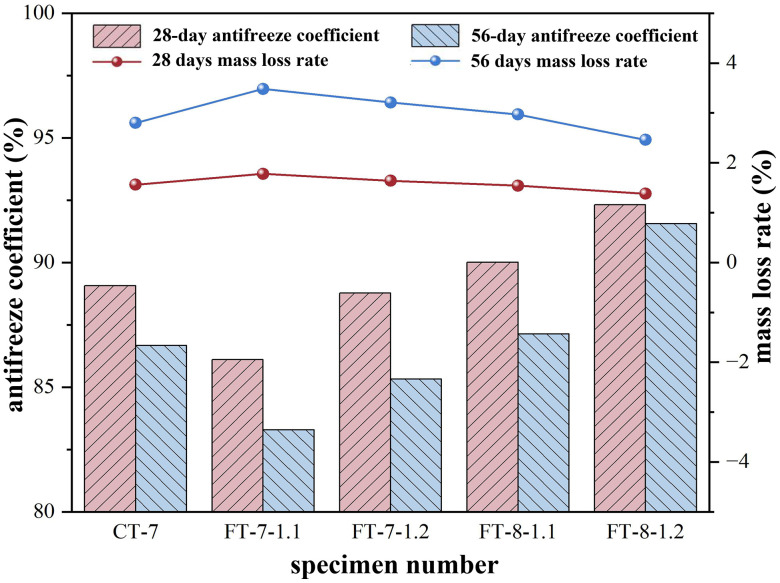
Changes in Frost Resistance and Mass Loss under Freeze-Thaw Cycles.

According to Figs 9 and 10, raising the binder content from 7% to 8% reduced mass loss and increased the freeze–thaw coefficient under both five and ten cycles. Strength retention mirrored these coefficient changes, confirming that higher binder contents better resisted freeze–thaw damage. This improvement was attributed to denser matrices, a refined pore network, and a strengthened interfacial transition zone that limited ice formation in critical pores and reduced hydraulic pressure during thaw (Table 15).

**Table 15:** Test Results of 5 Freeze-Thaw Cycles.

Serial Number	CT-7		FT-7–1.1		FT-7–1.2		FT-8–1.1		FT-8–1.2	
Cycles	5	10	5	10	5	10	5	10	5	10
Mass of Specimen before Freeze-Thaw (g)	6323	6321	6382	6379	6388	6382	6462	6456	6468	6464
Mass of Specimen after Freeze-Thaw (g)	6224	6144	6269	6157	6283	6177	6363	6265	6378	6305
Mass Loss Rate (%)	1.56	2.80	1.78	3.48	1.64	3.21	1.54	2.97	1.38	2.46
Compressive Strength before Freeze-Thaw (Mpa)	7.8	9.5	6.2	6.4	7.2	7.6	8.0	8.5	8.6	9.2
Compressive Strength after Freeze-Thaw (Mpa)	6.9	8.2	5.3	5.3	6.4	6.5	7.2	7.4	7.9	8.4
Frost Resistance Coefficient (%)	89.07	86.67	86.11	83.29	88.78	85.32	90.01	87.13	92.32	91.56

Increasing the activator dosage from 1.1% to 1.2% also improved the freeze–thaw coefficient and reduced mass variation at both binder levels and cycle counts, although the gains were smaller than those from increasing the binder. All freeze–thaw coefficients remained no less than 80 percent, indicating satisfactory frost resistance for base applications. Engineering implication: for cold-region subbases, allocating additional binder was generally more effective than increasing activator alone; excessive activator offered diminishing returns and could elevate shrinkage or efflorescence risks. The freeze–thaw durability coefficients were no less than the required limit across five and ten cycles, satisfying the frost-resistance requirement for base applications in JTG/T F20-2015.

### 4.5 Benchmarking against alternative AA-GS and commercial stabilizers

Under ambient curing, the AA-GS developed in this study attained 28-day compressive and flexural strengths of 46.2 MPa and 6.9 MPa, respectively, while also achieving water-stability coefficients≥0.876 and freeze-thaw durability indices≥0.80 in the subbase application window. Low-calcium fly-ash geopolymers often relied on heat assistance to achieve comparable strengths; ambient-cured variants tended to exhibit slower early-age kinetics and showed higher sensitivity to moisture/curing protocols. Cement-lime stabilizers remained the incumbent baseline in practice but generally showed lower early-age strength gain and higher water-sensitivity in coarse-grained spoil unless higher dosages or extended curing were used. In contrast, AA-GS leveraged calcium-rich slag and reactive aluminosilicates in coal gangue together with minor phosphogypsum to promote C-(A)-S-H/N-A-S-H gel co-formation under ambient conditions.

To contextualize the proposed alkali-activated gangue-slag (AA-GS) binder for subbase stabilization, we benchmarked our results against published envelopes for fly-ash-based and slag/fly-ash blended AA-GS under ambient curing, as well as commonly used commercial stabilizers (S6 File).

### 4.6 Preliminary cost-effectiveness assessment

We assembled a bill-of-materials (BoM) per m³ from the optimized AA-GS mix used in this study (gangue:slag = 1:1, activator dosage 14%, sodium-silicate modulus 0.8, L/S = 0.38) and from the cement–lime control used in practice. The total direct materials cost was evaluated by summation of unit-price–weighted quantities. Under the base procurement set (S7 File), AA-GS remained cost-comparable to the cement–lime baseline while delivering the mechanical and durability. The cost share analysis indicated that activators (sodium silicate and NaOH) dominated the AA-GS binder cost, whereas gangue and slag contributed modestly due to waste-sourced or low-grade industrial streams. A one-way sensitivity on the activator unit prices showed that moderate price reductions or partial substitution strategies (e.g., modulus tuning with sodium carbonate and minor Ca(OH)_2_ adjustment to maintain pH) preserved cost parity with negligible performance penalties in the tested window. Moreover, avoided disposal/tipping of gangue/tunnel spoil, when applicable, improved the net economics at project level even without altering the materials market prices. Direct material cost: AA-GS ≈ 52.6 CNY/m³; cement baseline≈56.0 CNY/m³.

### 4.7 Scalability and deployment considerations

To support scale-up beyond laboratory batches, we codified material-sourcing, plant operations, and field QC within the mix envelope validated in this study (silicate modulus 0.8, activator 14% at paste scale; 4–8% binder powder or 0.6–1.5% activator at the subbase scale; ambient curing).

(a) Feedstock variability (coal gangue, slag, minor phosphogypsum). Coal-gangue mineralogy and slag basicity varied across sources. We therefore adopted (i) lot-based XRF/XRD screening to track CaO, SiO_2_, Al_2_O_3_, Fe_2_O_3_, and SO_3_; (ii) pre-blending of multiple lots to dampen short-term excursions; and (iii) fineness matching to the study’s powder envelope. When SO_3_ or free-lime indications trended high, the corrective path prioritized (1) keeping the silicate modulus at 0.8 (to avoid unnecessary activator escalation) and (2) minor sulfate balancing via the existing phosphogypsum window, both within the ranges already validated. (b) Tunnel-spoil heterogeneity (moisture and gradation). Subbase performance depended on compaction relative to OMC/MDD established by Proctor tests. At plant scale we controlled the spoil moisture within a tight band around OMC using on-site moisture checks and metered addition of mixing water. Where the fines content deviated, we restored the target grading by selective screening/blending, keeping the binder dosage window (4–8% powder or 0.6–1.5% activator) unchanged. (c) Plant operations and energy. Deployment relied on ambient curing—no thermal curing or autoclaving was required—so energy was confined to standard mixing, pumping, and compaction. The dominant upstream burden remained the activator manufacturing. To mitigate it, we retained a low silicate modulus (0.8), avoided over-dilution (to limit solution mass), and considered partial carbonate activation and waste-derived silicate streams where regionally available, all without leaving the performance window validated here. (d) Field QC and acceptance. We defined acceptance as: (i) mix compliance—modulus 0.8 ± 0.05, activator dose within the subbase window; (ii) moisture/compaction—field density ≥ 98% of laboratory MDD with moisture controlled around OMC; and (iii) early checks—7-day UCS screening and water-stability index trending toward the 28-day targets. If out-of-window signatures were detected, S8 File listed corrective actions (e.g., minor activator tune, moisture re-balance, or fines adjustment). This operational envelope kept the scale-up path aligned with the laboratory-validated mixtures.

## 5 Conclusions

(1) Performance. Under ambient curing, AA-GS showed strong early development and stable later-age capacity, with lower drying shrinkage than the cement control and robust water and freeze–thaw durability. SEM revealed continuous gel formation and ITZ densification, which underpinned these strength and durability outcomes; overall performance met typical requirements for road-base applications without special curing.
(2) Environmental benefit. By valorizing locally available coal gangue and slag (with minor phosphogypsum), reducing clinker demand, and avoiding heat curing, AA-GS supported lower embodied carbon and dual-waste utilization.
(3) Field feasibility. The mix design and dosing protocol were compatible with routine road-base batching and compaction using readily sourced materials and ambient curing, supporting deployment—particularly in regions with limited access to premium fly ash.

## Supporting information

S1 File Abbreviations. (PDF)

S2 File Results of Dry Shrinkage Amount. (PDF)

S3 File Results of Dry Shrinkage Strain. (PDF)

S4 File Results of Water Loss Rate. (PDF)

S5 File Results of Dry Shrinkage Coefficient. (PDF)

S6 File Qualitative benchmarking of AA-GS against alternative AAMs and commercial stabilizers (ambient curing focus). (PDF)

S7 File Bill-of-materials and unit prices used for the direct materials cost calculation. (PDF)

S8 File Scale-up risk register and corrective actions (ambient deployment; consistent with the validated mix envelope). (PDF)
