# Supplementary material for: Experimental performance study on alkali-activated coal gangue-slag gel stabilized spoil for road base preparation
Source: PLoS One. 2026 Mar 31;21(3):e0343272. doi: 10.1371/journal.pone.0343272 (PMC13038017; doi:10.1371/journal.pone.0343272)
Supplement: S1 File — (PDF) [file pone.0343272.s001.pdf]

An abbreviation list was created to explain all the abbreviated phrases in the text.

File 1. Abbreviations

AA-GS	Alkali-Activated Gangue-Slag
AAMs	Alkali-Activated Materials
XRD	X-ray Diffraction
XRF	X-ray Fluorescence
GB	Guobiao (Chian)
JTG	Jiaotong Tujian Guifan (China)
CT	Cement-Stabilized Tunnel-Excavated Spoil
FT	Alkali-Activated Cement-Stabilized Tunnel Slag
UCS	Unconfined Compressive Strength
C-S-H	Calcium Silicate Hydrate
N-A-S-H	Sodium Aluminosilicate Hydrate
C-A-S-H	Calcium Aluminosilicate Hydrate
C-H-S	Calcium Hydroxide Silicate
C-H-F	Calcium Hydroxide Ferrite
C-H-A	Calcium Hydroxide Aluminate
