# Supplementary material for: Experimental performance study on alkali-activated coal gangue-slag gel stabilized spoil for road base preparation
Source: PLoS One. 2026 Mar 31;21(3):e0343272. doi: 10.1371/journal.pone.0343272 (PMC13038017; doi:10.1371/journal.pone.0343272)
Supplement: S2 File — (PDF) [file pone.0343272.s002.pdf]

The data in Supplementary File S_2 are the Results of Dry Shrinkage Amount, which correspond to the original data in Fig. 6A.

File 2 Results of Dry Shrinkage Amount

Age（d）	Dry Shrinkage Amount（mm）				
	CT-7	FT-7-1.1	FT-7-1.2	FT-8-1.1	FT-8-1.2
1	0.001	0.002	0.005	0.009	0.008
2	0.015	0.010	0.014	0.019	0.018
3	0.028	0.017	0.022	0.028	0.027
4	0.039	0.024	0.030	0.037	0.036
5	0.048	0.030	0.037	0.045	0.044
6	0.058	0.035	0.044	0.053	0.052
7	0.067	0.039	0.051	0.060	0.060
9	0.076	0.045	0.056	0.066	0.066
11	0.084	0.051	0.061	0.073	0.072
13	0.091	0.057	0.065	0.078	0.077
15	0.096	0.062	0.070	0.082	0.083
17	0.103	0.066	0.075	0.086	0.089
19	0.108	0.069	0.079	0.090	0.094
21	0.115	0.071	0.083	0.093	0.099
23	0.120	0.076	0.085	0.095	0.102
25	0.125	0.079	0.088	0.098	0.106
27	0.129	0.082	0.090	0.100	0.109
29	0.132	0.086	0.092	0.102	0.112
31	0.137	0.089	0.094	0.103	0.114
50	0.164	0.098	0.107	0.120	0.133

70	0.170	0.104	0.114	0.131	0.141
90	0.177	0.112	0.119	0.136	0.147
