# Supplementary material for: Experimental performance study on alkali-activated coal gangue-slag gel stabilized spoil for road base preparation
Source: PLoS One. 2026 Mar 31;21(3):e0343272. doi: 10.1371/journal.pone.0343272 (PMC13038017; doi:10.1371/journal.pone.0343272)
Supplement: S3 File — (PDF) [file pone.0343272.s003.pdf]

The data in Supplementary File S_3 are the Results of Dry Shrinkage Strain, which correspond to the original data in Fig. 6C.

File 3 Results of Dry Shrinkage Strain

Age（d）	Dry Shrinkage Strain（10^{-6}）				
	CT-7	FT-7-1.1	FT-7-1.2	FT-8-1.1	FT-8-1.2
1	2.5	5.0	12.5	22.5	20.0
2	36.3	25.0	35.0	47.5	45.0
3	70.0	42.5	55.0	70.0	67.5
4	96.3	60.0	75.0	92.5	90.0
5	120.0	75.0	92.5	112.5	110.0
6	143.8	87.5	110.0	132.5	130.0
7	167.5	97.5	127.5	150.0	150.0
9	188.8	112.5	140.0	165.0	165.0
11	210.0	127.5	152.5	182.5	180.0
13	226.3	142.5	162.5	195.0	192.5
15	240.0	155.0	175.0	205.0	207.5
17	256.3	165.0	187.5	215.0	222.5
19	270.0	172.5	197.5	225.0	235.0
21	286.3	177.5	207.5	232.5	247.5
23	300.0	190.0	212.5	237.5	255.0
25	311.3	197.5	220.0	245.0	265.0
27	322.5	205.0	225.0	250.0	272.5
29	328.8	215.0	230.0	255.0	280.0
31	342.5	222.5	235.0	257.5	285.0
50	408.8	245.0	267.5	300.0	332.5

70	425.0	260.0	285.0	327.5	352.5
90	441.3	280.0	297.5	340.0	367.5
