# Supplementary material for: Experimental performance study on alkali-activated coal gangue-slag gel stabilized spoil for road base preparation
Source: PLoS One. 2026 Mar 31;21(3):e0343272. doi: 10.1371/journal.pone.0343272 (PMC13038017; doi:10.1371/journal.pone.0343272)
Supplement: S4 File — (PDF) [file pone.0343272.s004.pdf]

The data in Supplementary File S_4 are the Results of Dry Shrinkage Strain, which correspond to the original data in Fig. 6B.

File 4 Results of Water Loss Rate

Age（d）	Water Loss Rate (%)				
	CT-7	FT-7-1.1	FT-7-1.2	FT-8-1.1	FT-8-1.2
1	1.11	0.70	1.19	1.41	0.91
2	1.43	1.30	1.69	1.93	1.36
3	1.94	1.74	1.94	2.46	1.85
4	2.46	2.12	2.12	2.69	2.12
5	2.91	2.30	2.18	2.73	2.48
6	3.24	2.48	2.46	2.88	2.75
7	3.48	2.63	2.77	3.16	3.06
9	3.71	2.75	2.91	3.31	3.20
11	3.90	2.84	3.00	3.39	3.29
13	4.09	3.11	3.21	3.57	3.48
15	4.24	3.23	3.38	3.73	3.63
17	4.38	3.36	3.54	3.88	3.78
19	4.47	3.45	3.66	3.99	3.89
21	4.54	3.52	3.76	4.08	3.99
23	4.61	3.59	3.85	4.04	4.08
25	4.68	3.66	3.89	4.10	4.19
27	4.76	3.74	3.91	4.11	4.30
29	4.80	3.78	3.82	4.17	4.32
31	4.84	3.83	3.87	4.20	4.38
50	4.88	3.87	3.93	4.21	4.44

70	4.91	3.90	4.08	4.29	4.47
90	4.98	4.03	4.11	4.38	4.51
