# Supplementary material for: Experimental performance study on alkali-activated coal gangue-slag gel stabilized spoil for road base preparation
Source: PLoS One. 2026 Mar 31;21(3):e0343272. doi: 10.1371/journal.pone.0343272 (PMC13038017; doi:10.1371/journal.pone.0343272)
Supplement: S5 File — (PDF) [file pone.0343272.s005.pdf]

The data in Supplementary File S_5 are the Results of Dry Shrinkage Strain, which correspond to the original data in Fig. 6D.

File 5 Results of Dry Shrinkage Coefficient

Age（d）	Dry Shrinkage Coefficient				
	CT-7	FT-7-1.1	FT-7-1.2	FT-8-1.1	FT-8-1.2
1	2.26	7.18	10.55	16.00	21.95
2	25.30	19.26	20.70	24.62	33.14
3	36.02	24.47	28.33	28.51	36.42
4	39.18	28.30	35.42	34.40	42.39
5	41.27	32.64	42.40	41.16	44.34
6	44.41	35.29	44.80	45.94	47.28
7	48.08	37.12	46.04	47.53	48.99
9	50.90	40.85	48.11	49.80	51.61
11	53.91	44.85	50.78	53.81	54.76
13	55.26	45.86	50.56	54.61	55.33
15	56.64	47.92	51.81	54.99	57.15
17	58.50	49.09	52.94	55.34	58.81
19	60.47	50.07	54.02	56.39	60.39
21	63.05	50.39	55.19	56.94	62.04
23	65.11	52.90	55.13	58.83	62.52
25	66.45	53.93	56.55	59.79	63.30
27	67.71	54.86	57.51	60.83	63.35
29	68.44	56.88	60.29	61.20	64.76
31	70.75	58.08	60.67	61.36	65.06
50	83.79	63.32	68.03	71.20	74.94

70	86.56	66.67	69.78	76.31	78.88
90	88.66	69.41	72.36	77.60	81.42
