# Supplementary material for: Experimental performance study on alkali-activated coal gangue-slag gel stabilized spoil for road base preparation
Source: PLoS One. 2026 Mar 31;21(3):e0343272. doi: 10.1371/journal.pone.0343272 (PMC13038017; doi:10.1371/journal.pone.0343272)
Supplement: S6 File — (PDF) [file pone.0343272.s006.pdf]

The engineering application results of the fly-ash-based and slag/fly-ash blended alkali-activated cementitious materials compared in this study are shown below.

File 6. Qualitative benchmarking of AA-GS against alternative AAMs and commercial stabilizers (ambient curing focus)

Dimension	AA-GS	Fly-ash-based AAM	Slag/Fly-ash	Commercial stabilizer
Early-age strength (≤7 d)	High (rapid gain)	Low–Moderate	Moderate–High	Low–Moderate
28-day strength (ambient)	High (≈46 MPa; 6.9 MPa flexural)	Moderate (often needs heat assistance)	High	Moderate
Water stability (subbase)	High (coeff. ≥0.876)	Moderate (protocol-sensitive)	High	Moderate (dosage-dependent)
Freeze–thaw resistance	High (index ≥0.80)	Moderate	High	Moderate
Curing practicality	Ambient; no heat	Often benefits from heat	Ambient or mild heat	Ambient
Activator dosage sensitivity	Moderate	Moderate–High	Moderate	Low
Moisture sensitivity	Low–Moderate	Moderate–High	Moderate	Moderate
Feedstock availability	High (gangue/slag widespread locally)	Declining/high-quality FA may be scarce	Variable	High
Field deployability	High	Moderate (protocol-sensitive)	High	High
