# Supplementary material for: Experimental performance study on alkali-activated coal gangue-slag gel stabilized spoil for road base preparation
Source: PLoS One. 2026 Mar 31;21(3):e0343272. doi: 10.1371/journal.pone.0343272 (PMC13038017; doi:10.1371/journal.pone.0343272)
Supplement: S7 File — (PDF) [file pone.0343272.s007.pdf]

The bill-of-materials (BoM) per cubic meter established in this study is shown below.

File7. Bill-of-materials and unit prices used for the direct materials cost calculation

Item	Symbol	Quantity per m^3 (q_i)	Unit	Unit price (p_i)
Coal gangue (powder)	q_{CG}	71.08	kg	100 CNY/t
Ground granulated slag	q_{GGBS}	71.08	kg	190 CNY/t
Sodium silicate solution	$q_{SS,sol}$	18.33	kg (as solution)	850 CNY/t
NaOH (pellets/solution)	Q_{NaOH}	6.04	kg (as solids)	3500 CNY/t
Mixing water	Q_{H2O}	201.15	kg (≈ 0.201 m^3)	2.6 CNY/m^3
Cement (baseline)	Q_{cem}	142.04	kg	390 CNY/t
Lime (baseline)	Q_{lime}	0.00	kg	715 CNY/t

Computation:

$C_{AA-GS} = \sum q_i p_i \,(AA - GS\ entries)$; $C_{cement} = \sum q_i p_i \,(baseline\ entries)$. A one-way sensitivity on P_{SS} and P_{NaOH} was reported in text. Unit prices reflect the current procurement cycle for the study region and can be updated by users to reproduce site-specific costs.

The AA - GS is approximately 52.6 CNY/m^3; the cement baseline is approximately 56.0 CNY/m^3, calculated using the formula.

$$C_{tot} = \sum_i q_i \cdot p_i$$
