# Supplementary material for: Experimental performance study on alkali-activated coal gangue-slag gel stabilized spoil for road base preparation
Source: PLoS One. 2026 Mar 31;21(3):e0343272. doi: 10.1371/journal.pone.0343272 (PMC13038017; doi:10.1371/journal.pone.0343272)
Supplement: S8 File — (PDF) [file pone.0343272.s008.pdf]

This study has integrated the risk register of the material in its scale-up application.

File 8. Scale-up risk register and corrective actions (ambient deployment; consistent with the validated mix envelope)

**Risk / Failure mode**	**Observable (screening)**	**Acceptance window (consistent with study)**	**Likely cause at scale**	**Corrective action (kept within validated ranges)**
Gangue/slag oxide drift (e.g., CaO ↑, SO3_3 ↑)	Lot XRF/XRD trend	Within historical band for the source; SO_3 not trending beyond internal baseline	Source variability; quarry change	Pre-blend lots; keep silicate modulus at **0.8**; adjust minor phosphogypsum within tested window; avoid activator escalation
Powder fineness off-spec	Blaine/laser PSD	±10% of study fineness envelope	Milling/screening drift	Restore fineness by screening or milling; maintain binder dosage window (**4–8%** powder)
Spoil moisture off OMC	Rapid moisture check	OMC ± **1–2%**	Weather, stockpile exposure	Metered water; cover stockpiles; re-check density vs MDD
Grading drift (fines ↑/↓)	Sieve analysis	Target gradation band restored	Excavation layer change	Blend with adjacent stockpile; light screening; keep activator 0.6–1.5%
Early-age under-strength	7-d UCS screen	Trending toward 28-d target	Low temperature; under-compaction; overdilution	Ensure density ≥98% MDD; trim solution water; **do not** raise modulus above 0.8 unless outside tolerance
Efflorescence /	Visual, mass	Cosmetic only;	Over-alkalinity at	Reduce surface water;

Risk / Failure mode	Observable (screening)	Acceptance window (consistent with study)	Likely cause at scale	Corrective action (kept within validated ranges)
surface salt	loss	no mass loss	surface; wet curing	maintain low modulus; brief cover curing
Mixing energy / plant uptime	kWh/m³ log	Stable; no heat load	Improper batching; pump shear	Calibrate batching; avoid unnecessary recirculation

Note: All actions **respected** the validated mix envelope (modulus 0.8; activator 14% paste; 4–8% powder or 0.6–1.5% activator at subbase scale; ambient curing).
